# ICD Outcome in Pediatric Cardiomyopathies

**DOI:** 10.3390/jcdd9020033

**Published:** 2022-01-20

**Authors:** Massimo Stefano Silvetti, Ilaria Tamburri, Marta Campisi, Fabio Anselmo Saputo, Ilaria Cazzoli, Nicoletta Cantarutti, Marianna Cicenia, Rachele Adorisio, Anwar Baban, Lucilla Ravà, Fabrizio Drago

**Affiliations:** 1 Pediatric Cardiology and Cardiac Arrhythmia/Syncope Unit, Bambino Gesù Children’s Hospital IRCCS, 00165 Rome, Italy; ilaria.tamburri@opbg.net (I.T.); marta.campisi@opbg.net (M.C.); fabioabnselmo.saputo@opbg.net (F.A.S.); ilaria.cazzoli@opbg.net (I.C.); nicoletta.cantarutti@opbg.net (N.C.); marianna.cicenia@opbg.net (M.C.); rachele.adorisio@opbg.net (R.A.); anwar.baban@opbg.net (A.B.); fabrizio.drago@opbg.net (F.D.); 2 Epidemiology Institute, Bambino Gesù Children’s Hospital IRCCS, 00165 Rome, Italy; lucilla.rava@opbg.net

**Keywords:** implantable cardioverter defibrillator (ICD), cardiomyopathies, sudden cardiac death, pediatric age

## Abstract

Background: Pediatric patients with cardiomyopathies are at risk of malignant arrhythmias and sudden cardiac death (SCD). An ICD may prevent SCD. The aim of this study was to evaluate ICD implantation outcomes, and to compare transvenous and subcutaneous ICDs (S-ICDs) implanted in pediatric patients with cardiomyopathies. Methods: The study is single center and retrospective, and includes pediatric patients with cardiomyopathies who required ICD implantation (2010–2021). Outcomes were recorded for appropriate/inappropriate ICD therapy and surgical complications. Transvenous ICD and S-ICD were compared. Data are presented as median values (25th–75th centiles). Results: Forty-four patients with cardiomyopathies (hypertrophic 39%, arrhythmogenic 32%, dilated 27%, and restrictive 2%) underwent transvenous (52%) and S-ICD (48%) implantation at 14 (12–17) years of age, mostly for primary prevention (73%). The follow-up period was 29 (14–60) months. Appropriate ICD therapies were delivered in 25% of patients, without defibrillation failures. Lower age at implantation and secondary prevention were significant risk factors for malignant ventricular arrhythmias that required appropriate ICD therapies. ICD-related complications were surgical complications (18%) and inappropriate shocks (7%). No significant differences in outcomes were recorded, either when comparing transvenous and S-ICD or comparing the different cardiomyopathies. Conclusions: In pediatric patients with cardiomyopathy, ICD therapy is effective, with a low rate of inappropriate shocks. Neither ICD type (transvenous and S-ICDs) nor the cardiomyopathies subgroup revealed divergent outcomes.

## 1. Introduction

Cardiomyopathies in pediatric patients involve the risk of malignant arrhythmias that may cause sudden cardiac death (SCD) [1,2,3,4]. In the National Australian Childhood Cardiomyopathy Study, the cumulative incidence of SCD at 15 years was 5% for dilated cardiomyopathy (DCM), 6% for hypertrophic cardiomyopathy (HCM), 12% for restrictive cardiomyopathy (RCM), and 23% for left ventricular (LV) noncompaction [2]. Specific data regarding SCD in childhood onset arrhythmogenic cardiomyopathy (ACM) are difficult to find due to limited study and observation of this condition in this cohort.

ICD may prevent SCD from malignant ventricular arrhythmias in children with cardiomyopathies [5,6,7], increasing the number of ICDs implanted for primary prevention [8]. However, in this specific cohort, several factors may affect the use of ICD, causing complications related mainly to physiologic parameters in children (body dimensions, progressive growth both in height and weight, complex anatomy, physical activity) and devices (size, lead characteristics, implantation approach and procedure) [9]. These complications can reach as high as 20–30% for inappropriate shocks and 39% for surgical complications [10,11]. A new ICD system, the entirely subcutaneous ICD (S-ICD) without endovascular or epicardial leads, has been developed and implanted in pediatric patients and this has showed its efficacy at terminating ventricular arrhythmias [12,13,14]. The aim of this study was to evaluate the outcome for pediatric patients with cardiomyopathies in a single tertiary pediatric center who had ICDs implanted, and to compare the outcome of both transvenous ICD and S-ICD in this population.

## 2. Materials and Methods

The study is single centered and retrospective, including pediatric patients with cardiomyopathies who required ICD implantation for SCD prevention. Enrolled patients included pediatric patients followed-up at the Cardiomyopathy Unit of Bambino Gesù Children’s Hospital IRCCS who underwent ICD implantation from January 2010 to January 2021 at the Pediatric Cardiac Arrhythmias Unit. Inclusion criteria were: (i) age ≤ 18 years (ii), the presence of a cardiomyopathy, and (iii) an ICD implanted following current indications (see Section 2.1). Exclusion criteria were: (i) patients with ICD implanted for other disease (congenital, channelopathy), and (ii) patients with epicardial ICD. Data were collected at in-hospital follow-up evaluations and during remote-monitoring analysis. All data were registered and retained in the hospital’s computing archives. Recorded data contained demographic and procedural data, data concerning cardiac diagnosis and clinical severity, appropriate ICD therapy, issues with complications, and clinical status at most recent follow-up evaluation. This study complies with the Declaration of Helsinki and was approved by the local ethics committee. Informed consent was obtained from the guardians of all patients.

### 2.1. Indications for ICD Implantation

ICDs were implanted in accordance with the current guidelines/consensus statement [7,15,16,17,18].

Primary prevention was considered to be a class II indication for HCM and chronic optimal medical therapy with two or more major risk factors: family history of SCD (one or more first-degree relatives with SCD aged < 40 years with or without an HCM diagnosis, or SCD in a first-degree relative at any age with an established diagnosis of HCM); ≥1 episode of unexplained, recent syncope; massive LV hypertrophy (maximum left ventricular wall thickness ≥ 30mm or a Z-score ≥ 6); non-sustained ventricular tachycardia (VT) [15,16]. The presence of late gadolinium enhancement during cardiac magnetic imaging was considered to be an additional risk factor [3].

Similarly, increased risk for SCD in ACM was linked to the presence of sustained VT, extensive right and LV involvement, and SCD in family members with ACM. As a prophylactic, primary prevention, ICD implantation was recommended in patients with severe right and/or LV dysfunction, syncope, and non-sustained VT. Furthermore, moderate right and/or LV dysfunction, were considered ‘major’ risk factor that led to a prophylactic ICD implantation [15,17].

Regarding DCM, pediatric recommendations were embedded within adult studies for indication of ICD delivery in patients with nonischemic DCM. The criteria included LV ejection fraction (EF) < 30–35%, New York Heart Association functional class II or III, along with age at diagnosis < 14 years [4,18].

In patients with a history of sustained VT and resuscitated ventricular fibrillation (VF), ICD implantation was due to secondary prevention.

### 2.2. Implantation Procedure, ICD, and Leads

The indications and implantation procedure for transvenous and subcutaneous systems have been previously described in detail [9,13,19]. Briefly, transvenous implantations were performed in patients with a body weight ≥30 kg by accessing the subclavian/axillary vein. The transvenous leads were positioned in the right atrium and right ventricle (RV) apex. Leads were implanted in the presence of good pacing (<1 V/0.5 ms), sensing (atrium > 1 mV, ventricle > 5 mV), and impedance values (<1000 ohms). Transvenous leads were fixed to subcutaneous tissues with absorbable ligatures in children, and with non-absorbable ligatures in post-puberty patients. Single (VVI) and dual (DDD) chamber devices were implanted: VVI devices were implanted in smaller patients and in those not requiring atrial pacing; DDD devices were implanted in older patients, in those requiring atrial pacing (as in patients with bradycardia induced by antiarrhythmic agents), and in patients with supraventricular arrhythmias who needed atrial anti-tachycardia pacing. Single and dual coil leads were used at the discretion of the electrophysiologist. The ICD was positioned above (pre-pectoral pocket) or below (sub-pectoral pocket) the pectoralis major muscle according to the patient’s dimensions and the physician’s discretion.

The S-ICDs were implanted in patients: (i) older than 7–8 years of age, (ii) with a body mass index (BMI) generally ≥ 20 kg/m^2^, (iii) who fulfilled screening criteria, and (iv) who did not require pacing [9,13,20]. A subcutaneous pocket was created over the fascial plane covering the “serratus anterior” muscle in the left lateral thoracic region in the proximity of the 5th and 6th intercostal spaces near the mid-axillary line [13]. For the intermuscular ICD location, the pocket was created between the “latissimus dorsi” and the “serratus anterior” muscles [21]. Subcutaneous defibrillation leads were implanted through a 3-incision (standard) or a 2-incision (simplified) procedure [22]. A small incision was made at the xiphoid process (a xiphoid incision), on the left or right side, according to the screening results. The electrode insertion tool was introduced into the xiphoid incision and the lead was tunneled laterally as far as the device pocket. In the standard procedure, a third small incision was made at the sterno-manubrial junction. The distal tip of the electrode was tunneled subcutaneously using the insertion tool towards the superior incision, where the lead tip was anchored with sutures. The simplified procedure differed from the previous one as it avoided the superior incision and suture. The lead, through the insertion tool and a peel-away sheath, was tunneled along the parasternal line up to the desired position at the sterno-manubrial junction.

A defibrillation test was performed at the end of the implantation procedure in all S-ICD patients, except for patients with EF ≤25%. A defibrillation test was not performed after transvenous ICD implantation.

All procedures were performed in the electrophysiology/cardiac pacing laboratory, which is also approved for surgical procedures. Device implantations were performed under general anesthesia by the team of pediatric electrophysiologists/anesthesiologists with the support of the manufacturer’s technicians, and of the pediatric cardiac surgeons, when required. Antibiotic prophylaxis was given to every patient according to our institution’s guidelines.

### 2.3. Follow-Up

Patients were followed-up at 1, 3, and 6 months, and then every 3–4 months, with ICD telemetric interrogation, ECG, and echocardiography evaluation performed. Remote monitoring was activated for all patients, except for patients with the first-generation S-ICD (1010 SQ-RX) devices, which were not enabled to remote monitoring. Patient-activated transmissions for remote monitoring were performed once a month by patients/parents. Device-activated transmissions were automatically performed in the presence of arrhythmias, electrical therapies, or device function abnormalities.

### 2.4. Statystical Analysis

Continuous variables are described as their median values (25th–75th centiles). Categorical variables are reported according to their absolute and relative frequencies. Outcomes between transvenous and S-ICD were compared. Appropriate shocks and/or effective anti-tachycardia pacing to treat malignant tachyarrhythmias, i.e., sustained VT or VF, were considered to be effective and appropriate ICD therapies. Device-related complications included complications requiring surgical revision (related to surgical wounds, device pocket, and lead) and inappropriate shocks. The difference between continuous variables was tested with the non-parametric Wilcoxon test. Categorical variables were compared using a chi-square test, as appropriate. Kaplan–Meier survival analysis was used to study freedom from adverse events. Survival curves were compared with the log-rank test. Cox regression analysis, both univariate and multivariate, was applied to verify possible event predictors. The hazard ratio (HR), the 95% confidence interval (95% CI), and relative significance were reported for each covariate in the model. A *p* < 0.05 was considered to be significant.

All analyses were performed with StataSE 12.0 (StataCorp, College Station, TX, USA).

## 3. Results

The study included 44 patients with cardiomyopathies who underwent ICD implantation at Bambino Gesù Children’s Hospital (Rome) from January 2010 to January 2021. The patients’ characteristics are reported in Table 1. The cardiomyopathy subtype varied, including HCM in 39%, ACM in 32%, DCM in 27%, and RCM in 2%.

In six of the seventeen patients, the HCM was obstructive.

There were four patients with syndromic HCM (23%): two had Noonan syndrome, one had LEOPARD syndrome, and one had Danon disease.

Six patients with DCM were affected by neuromuscular disease (50%): laminopathy was present in four, and Duchenne muscular dystrophy was present in two patients.

ICDs were implanted at 14 (12–17) years of age, mostly for primary prevention (73%). Secondary prevention was required in 12/44 (27%) for sustained VT (six cases) and VF (six cases, including the RCM patient). The devices’ characteristics are reported in Table 1 and Table 2.

Statistics for transvenous ICDs (52%) and S-ICDs (48%) are shown in Table 3.

Transvenous ventricular leads were single coil in 18 patients (78%), and they were dual coil in five patients (22%).

S-ICDs were preferred for primary prevention. The median BMI of these patients was 22 (20.5–26) kg/m^2^. S-ICDs were implanted in patients with a modified two-incision surgical technique in 19 patients. Only the first two patients, with ACM, underwent a standard three-incision technique.

A defibrillation test was performed at the end of the S-ICD implantation procedure in all patients, except for one DCM patient with an EF of 25%. Three S-ICD patients (14%) were not inducible, and only a test shock of 10 J was given to measure impedance. The defibrillation test was effective in all the remaining 17 patients. The defibrillation test was performed at 65 J in all but four patients, who were tested at a lower energy of 40 J. Sensing vectors included the primary (50% of cases), the secondary (40%), and the alternate (10%) vectors.

All implanted devices were enabled for remote monitoring, except the first three S-ICDs, which were 1010 SQ-RX models.

### 3.1. ICD Programming

Transvenous ICDs were programmed with two zones: VF, median 250 (220–250) bpm, and VT, median 220 (180–220) bpm. In all patients, anti-tachycardia pacing was programmed on.

The programmed shock zone for the S-ICD was 250 (200–250) bpm and the conditional zone was 210 (180–230) bpm.

### 3.2. Follow-Up

The follow-up was extended to October 2021. Its duration was 29 (14–60) months. The longest follow-up was 120 months in two patients. Some patients were lost at long-term follow-up: five underwent heart transplantation, one received a ventricular assistance device, and two were deceased following non-device related death (refractory heart failure).

### 3.3. ICD Therapies

**Figure 1:**
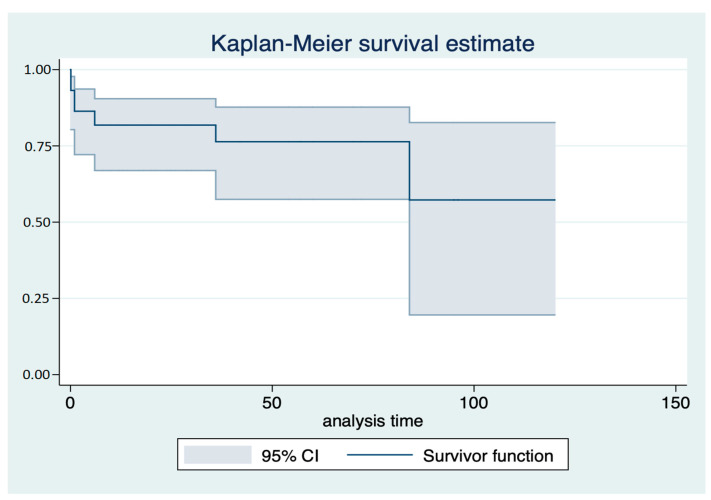
Kaplan–Meier survival estimates for freedom from effective and appropriate ICD therapy in the whole cohort. Analysis time: months.

Over the follow-up, 11 patients (25%) received appropriate ICD therapies after 12 (12–24) months (Table 1, Table 2 and Table 3). There were six appropriate shocks and five effective anti-tachycardia pacing treatments. No defibrillation failure occurred. Electrical storms were not recorded. Kaplan–Meier survival estimates showed freedom from ICD therapies in nearly 70% of patients at 50 months (Figure 1).

**Figure 2:**
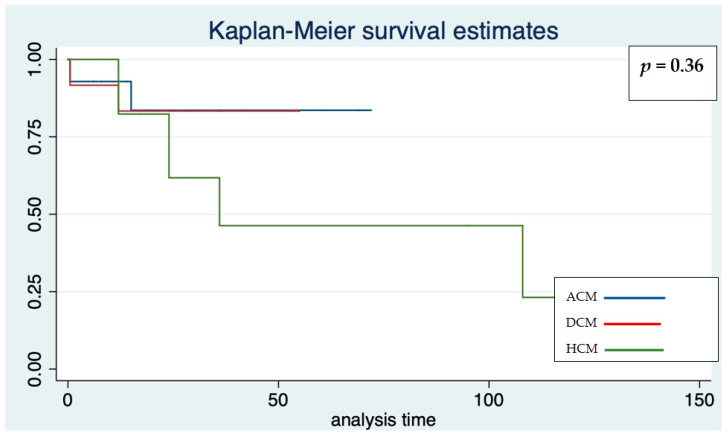
Kaplan–Meier survival estimates for freedom from effective and appropriate ICD therapy according to the three main cardiomyopathies in the whole cohort. Analysis time: months.

**Figure 3:**
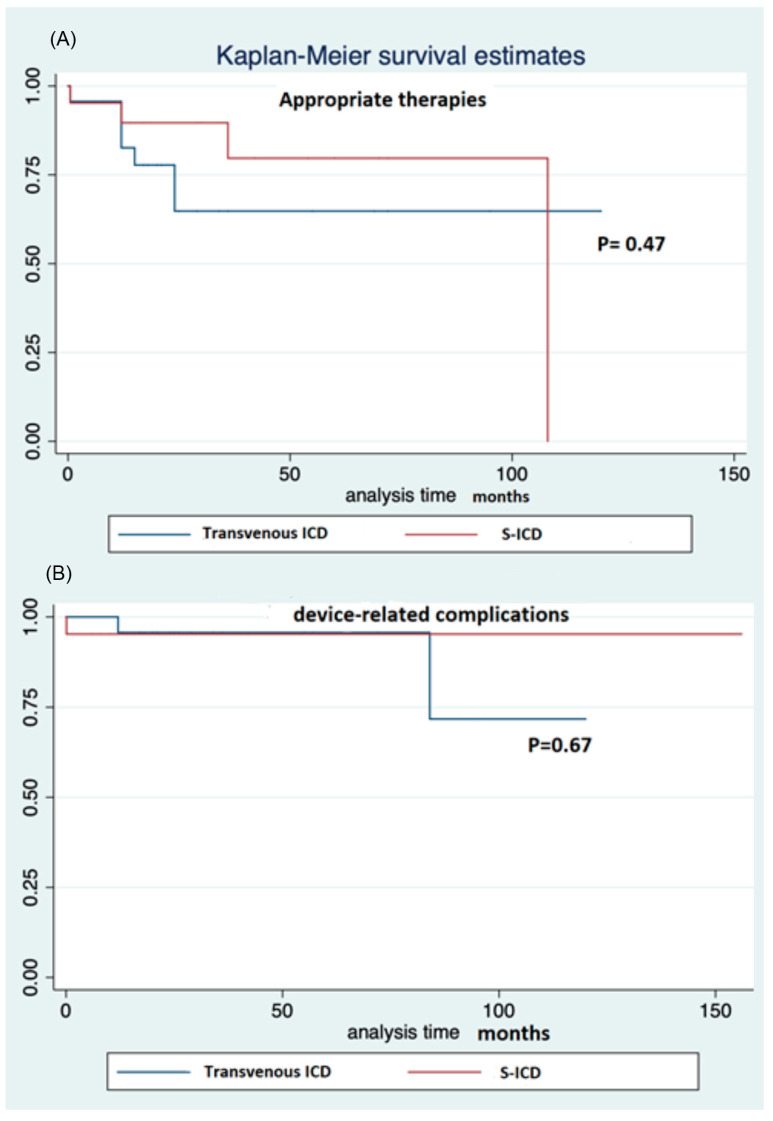
(**A**) Kaplan–Meier survival estimates for freedom from effective and appropriate ICD therapy according to type of ICD. (**B**) Kaplan–Meier survival estimates for freedom from ICD complications according to type of ICD.

There were no significant differences in the efficacy of ICD according to the type of cardiomyopathy (Figure 2, Table 2), the device system (seven effective therapies in transvenous, four in S-ICD, *p* = 0.47, Table 3, Figure 3), sex (five males, six females, *p* = 0.37), the presence of associated disease (one versus ten effective therapies in patients with or without associated disease, respectively, *p* = 0.15).

**Figure 4:**
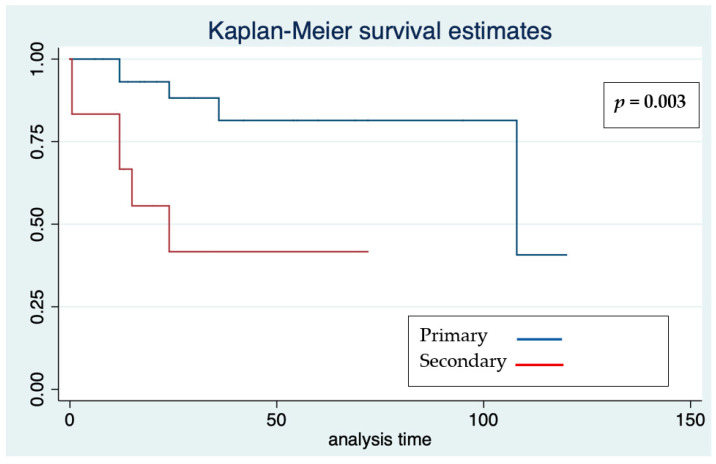
Kaplan–Meier survival estimates for freedom from effective and appropriate ICD therapy according to prevention in the whole cohort. Analysis time: months.

Significant differences occurred relating to primary and secondary prevention (Figure 4). Cox regression analysis showed that older age at implantation was significant for a 14% risk reduction of malignant arrhythmias and therefore appropriate therapies, with an HR of 0.86 (95% confidence interval 0.74–0.99. *p* = 0.038).

### 3.4. Device-Related Complications

**Figure 5:**
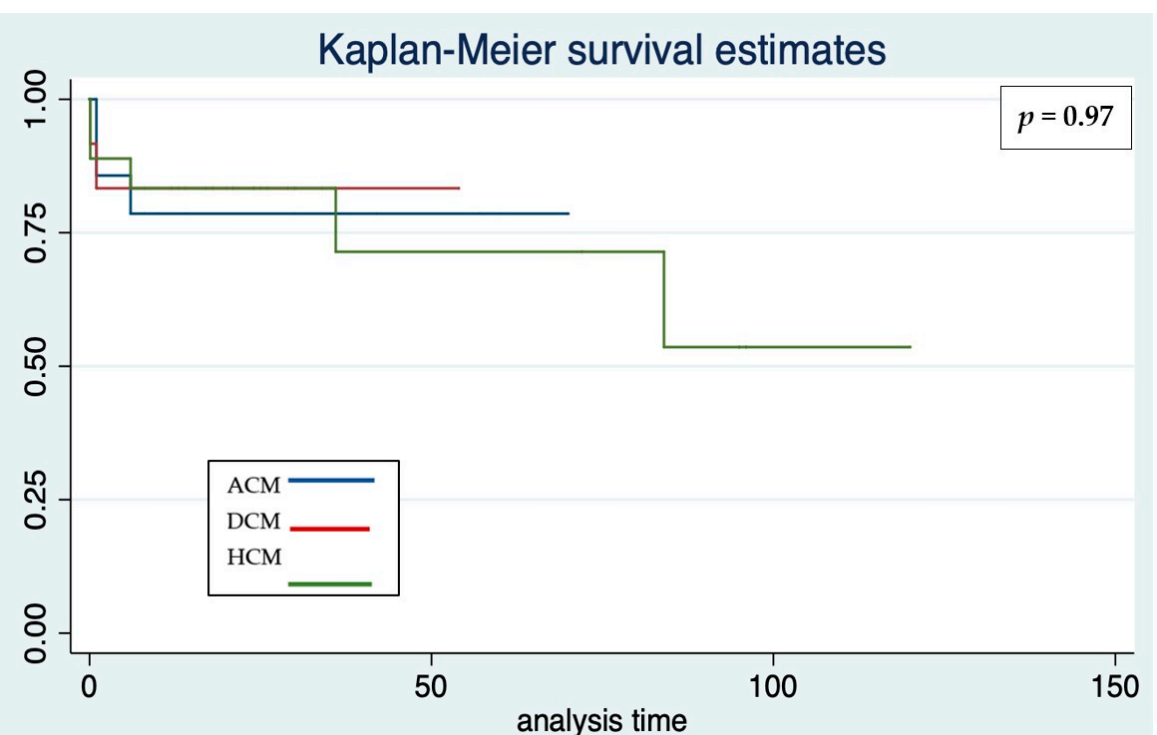
Kaplan–Meier survival estimates for freedom from ICD complications according to cardiomyopathy in the whole cohort. Analysis time: months.

ICD system-related complications occurred in 11 patients (25%). This included eight complications requiring surgical revision and three inappropriate shocks (Table 1, Table 2, Table 3 and Table 4). No significant differences were observed according to the type of cardiomyopathy (Table 2 and Figure 5), prevention (nine complications in primary prevention, and two in secondary prevention groups, *p* = 0.28), device system (six cases in transvenous, five in S-ICD groups), Table 3 and Figure 3), gender (four females and seven males, *p* = 0.28), associated disease (three complications in the presence of associated disease versus eight in its absence, *p* = 0.89), and age at implantation, with an HR of 0.92 (95% confidence interval 0.79–1.08, *p* = 0.30), either for total device-related complications, or for inappropriate shocks. One patient experienced both appropriate and inappropriate shocks (one of each).

Complications requiring surgical revision included four lead dislodgements (three transvenous, and one subcutaneous). All leads were repositioned. Additionally, in the S-ICD group, three device leads showed erosions: all underwent surgical revisions. Only in one patient was it not successful, and the system was therefore explanted. The last complication was a hemopericardium 3 days after the implantation of a dual-chamber ICD in a DCM patient. It was drained without further complications.

Inappropriate shocks occurred 6 (0.1–84) months after implantations. Two involved the transvenous systems for cardiac (T-wave) oversensing and high-rate supraventricular tachyarrhythmia exceeding the VF limit, and one involved an S-ICD for non-cardiac oversensing (entrapped air around the subcutaneous lead). Cardiac oversensing was solved by device reprogramming: the VF limit was increased, antiarrhythmic drug treatment was added, and subcutaneous air spontaneously disappeared (the S-ICD therapies were switched off during in-hospital stay until the air disappeared, as determined with a chest X-ray).

## 4. Discussion

This retrospective study aimed to compare the outcome of transvenous ICD and S-ICD in pediatric cardiomyopathies. Our results did not show significant differences in outcomes between the two subgroups. Most of the patients received an S-ICD that was chosen from among the latest produced models that are enabled to remote follow-up and were implanted using the newest surgical techniques (two-incision, intermuscular). In addition, ICD outcomes were not significantly different among the three main cardiomyopathy subgroups (HCM, ACM, and DCM), with a 25% overall efficacy of the ICD system. No defibrillation failure was observed among our patients. Lower age at implantation and secondary prevention were significant risk factors for the delivery of appropriate therapies due to the occurrence of arrhythmias. Few inappropriate shocks (7%) occurred. It is probable that this relevant result is due to the high-rate limit programming for VF and the recent technical improvements in manufacturing the devices: new systems (S-ICD) and new algorithms (such as the “smart pass” ™ in S-ICD) may reduce complications. Moreover, the new remote monitoring devices may lead to an early diagnosis of malfunctions and complications [23]. Complications occurred earlier than effective therapies. An inappropriate shock was observed early after S-ICD implantation due to entrapped subcutaneous air at the incision of the lead dipole. This complication led us to modify the post-implantation protocol: we started to wait until the first chest X-ray after the procedure excluded the presence of air bubbles along the lead or device to activate the S-ICD therapies.

In the pediatric population, the main ICD systems used are transvenous, epicardial devices with defibrillation coils implanted in the subcutaneous tissue, pericardial, or pleural space, and S-ICDs [9]. Over the last decade, the introduction of devices with remote monitoring and follow-up and the S-ICD have been two substantial changes to the everyday pacing practice that have increased the ICD performances and reduced related risks. In that period of time, several studies have enrolled 955 patients (aged 14 years) for primary prevention (53% of cases) of channelopathy-related arrhythmia (44%), cardiomyopathies (36%), congenital diseases (18%), and other diseases (2%). The ICDs implanted were transvenous systems (42%), epicardial + subcutaneous coil systems (23%), and S-ICDs (35%). The overall results showed that 28% experienced appropriate shocks, 20% experienced inappropriate shocks, and 23% experienced complications (including 5% with infections) after a follow-up period of 4–5 years [8,10,12,13,14,24,25,26,27,28].

Therefore, the present study has showed the overall efficacy of the ICD system comparable to previously published data, and fewer device-related complications than those reported in the literature data due to the low rate of inappropriate shocks.

Over the same period, few studies have focused on S-ICDs in pediatric and young-adult populations [12,13,14,29,30,31]. These six studies included 221 patients, aged 17 years on average, implanted for primary prevention (60%) of cardiomyopathies (30%), congenital disease (21%), channelopathies (44%), and other diseases (5%). Two-incision and intermuscular procedures were performed in 36% and 6% of cases, respectively. At the 3-year follow-up, the outcome revealed the experiences of appropriate (20%) and inappropriate shocks (15%), and surgical complications were reported in 13%. The S-ICD data from the present study could be comparable to the above results, but with a lower rate of inappropriate shocks that confirms the safety and effectiveness of S-ICDs. However, the device size seems to be still too large for small children and thin adolescents, and the need for ECG screening together with the lack of anti-tachycardia/anti-bradycardia pacing are further limitations. Nevertheless, these disadvantages are overcome by all the advantages of an S-ICD, with its simplified implantation procedure and device programming, the absence of endocardial or epicardial devices, its easier and risk-free extraction procedure, and the less harmful effects of shocks [20].

Most of the published pediatric studies regarding ICD in cardiomyopathies have focused on HCM. Moreover, the attempt to find specific data about cardiomyopathy subtypes from the cited literature only returned data about HCM. Dechert et al. studied a subgroup of 24 HCM patients aged 12 years and followed-up for nearly 5 years: they reported that 13% experienced appropriate therapies, 17% experienced inappropriate shocks, and 21% underwent implant revisions [10]. Kaski et al. [32] studied 22 patients (median age 14 years) with transvenous ICD in HCM, reporting that 18% experienced appropriate and inappropriate shocks, with 9% experiencing complications. Two multicenter studies reported on large numbers of HCM adolescent patients. One of these, by Maron et al. [33], described 224 HCM patients with transvenous (82%) and epicardial ICD, implanted for primary prevention in 84% of cases, who showed that 19% and 28% experienced appropriate and inappropriate shocks, respectively, and 12% experienced complications, including RV perforation, a lacerated coronary vein, and an exploratory sternotomy. The other was conducted by Kamp et al. [34], and described 73 HCM patients who underwent transvenous (96%) and epicardial ICD implantation for primary prevention (83%), and their findings were appropriate/inappropriate shocks in 11%/22%, respectively, while complications occurring in 32% of patients. A recent multicenter study on HCM [6] showed the administration of 28% appropriate and 8% inappropriate therapies, with 31% device-related (lead/infection) complications. Therefore, data from these studies on HCM could be comparable with those of the present study, as reported in Table 2.

In the literature, there are no data describing the outcome of ICDs implanted in ACM pediatric patients. A national Italian S-ICD multicenter registry including majorly ACM adult patients [35] showed 14% appropriate and inappropriate shocks, and 7% device-related complications.

Likewise, ICD outcomes in pediatric DCM are scarcely described. Some studies evaluated this parameter in DCM pediatric patients awaiting heart transplantation [36,37]. The overall results showed that the risk of SCD was not different among transplant candidates with or without ICD [36]. Moreover, the prophylactic use of the ICD in children with DCM and symptomatic heart failure did not appear to be cost effective, and this could be due to lower rate of SCD in this population [38].

Therefore, to our knowledge, the present study is the first report about ICD implantation outcomes in small ACM and DCM pediatric cohorts.

Some of our patients with DCM had neuromuscular disease [39]. As already reported, the use of an ICD for Duchenne muscular dystrophy cardiomyopathy may be associated with improved survival rates and minimal complications in patients who show severe LV dysfunction [40,41]. On the other hand, our cohort included patients with pediatric onset laminopathy, where further delineation was detailed in a separated report [42].

### Limitations

As in most pediatric studies, and also in the present single-center retrospective study, the number of patients included is small. This may be relevant in the subgroup analysis (type of cardiomyopathy, transvenous ICD, S-ICD, different outcomes), and hence caution must be paid for a robust comparison. Moreover, the follow-up may be too short to calculate the occurrence of appropriate shocks and all ICD system complications, since these parameters might be time related [19]. However, it was previously reported that the incidence of effective therapies and complications tend to show a decreasing trend over time [8]. Subsequently, due to the relatively short observation time and limited cohort size, longer and larger multicentric studies are still needed to confirm these results.

## 5. Conclusions

All pediatric patients with cardiomyopathies who underwent an ICD implantation since 2010 at our institution, mostly with remote monitoring-enabled devices and for primary prevention, showed a 25% rate of effective therapies and complications. There were no significant differences in outcomes between transvenous and subcutaneous ICDs, as well as between the three major subtypes of cardiomyopathies. There were no defibrillation failures. Lower age at implantation and secondary prevention were significant risk factors for malignant ventricular arrhythmias that required appropriate ICD therapies. Nearly 70% of patients showed freedom from ICD-appropriate therapies at around 4 years of follow-up. Most ICD-related complications required surgical revision (device pocket, leads) and there was a low rate (7%) of inappropriate shocks.

## Figures and Tables

**Table 1:** Patients’ and devices’ characteristics.

	Number (%)	Number (%) or Median (25th–75th Centiles)
Patients	44 (100%)	
Males	24 (54%)	
Age, years		14 (12–17)
Height, cm		164 (151–171)
Weight, kg		54 (44–69)
Hypertrophic cardiomyopathy	17 (39%)	
Arrhythmogenic cardiomyopathy	14 (32%)	
Dilated cardiomyopathy	12 (27%)	
Restrictive cardiomyopathy	1 (2%)	
Primary prevention	32 (73%)	
Secondary prevention	12 (27%)	
Transvenous ICD	23 (52%)	
VVI		12 (52%)
DDD		11 (48%)
Pre-pectoral pocket		16 (70%)
Sub-pectoral pocket		7 (30%)
Subcutaneous ICD	21 (48%)	
Subcutaneous pocket		9 (43%)
Intermuscular pocket		12 (57%)
Follow-up		29 (14–60) months
Appropriate therapies	11 (25%)	12 (12–24) months
Total complications	11 (25%)	1 (0.6–18) months
Complications requiring surgical revision	8 (18%)	1 (0.8–12) months
Inappropriate shocks	3 (7%)	6 (0.1–84) months

Data are given as number (%) or median (25th–75th centiles). See text for further details.

**Table 2:** Specific cardiomyopathies and ICD.

	Pts	Primary Prev.	Height at Impl. cm	Weight at Impl. kg	Age at Impl. yrs	Effective Therapy	Complications Requiring Surgical Revision	Inappropriate Shocks	Follow-Up, Mos.
HCM	17 39%	14 82%	152 (146–165)	48 (38–55)	12 (10–14)	7 41%	3 18%	2 12%	29 (17–91)
DCM	12 27%	8 67%	180 (166–180)	57 (44–72)	15 (13–17)	2 17%	3 25%	0	22 (13–31)
ACM	14 32%	10 71%	163 (155–171)	61 (50–71)	15 (14–17)	2 14%	2 14%	1 7%	49 (18–67)
RCM	1 2%	0	168	48	14	0	0	0	18

Data are given as number (%) or median (25th–75th centiles). Differences are not significant. Abbreviations: ACM: arrhythmogenic cardiomyopathy; DCM: dilated cardiomyopathy; HCM: hypertrophic cardiomyopathy; Impl.: implantation; Mos: months; Prev. prevention; Pts: patients; RCM: restrictive cardiomyopathy; yrs: years.

**Table 3:** Transvenous and subcutaneous ICDs.

ICD	Pts	Primary Prev.	Height at Impl. cm	Weight Impl. kg	Age Impl. yrs	Sub-Cutaneous Pocket	Effective Therapy	Complications Requiring Surgical Revision	Inappropriate Shocks	Follow-Up, Mos.
Trans-venous	23	13 56%	161 (146–180)	48 (38–67)	14 (12–17)	16 70%	7 30%	4 17%	2 9%	26 (19–63)
Sub-cutaneous	21	19 90%	164 (155–171)	58 (52–69)	14 (12–17)	9 43%	4 19%	4 19%	1 5%	30 (14–60)

Data are given as number (%) or median (25th–75th centiles). Differences are not significant. Abbreviations: see Table 2.

**Table 4:** Device-related complications.

Complications	n.	Time to Complication, Months	Device	Cardiomyopathy	Treatment
Inappropriate shock	3	6 (0.1–84)	2 TV-ICD 1 S-ICD	2 HCM 1 ACM	2 reprogramming (+1 drug treatment), none
Pocket/wound related	3	6 (3–21)	3 S-ICD	2 HCM 1 ACM	2 revision, 1 explant
Lead related	4	1 (0.7–8)	3 TV-ICD 1 S-ICD	2 DCM 1 ACM 1 HCM	Lead repositioning
Pericardial effusion	1	0.1	TV-ICD	DCM	Drainage

Data are given as number and median (25th–75th centiles). Abbreviations: ACM, DCM, and HCM: arrhythmogenic, dilated, and hypertrophic cardiomyopathy; S-ICD: subcutaneous ICD; TV-ICD: transvenous ICD. See text for further details.

## Data Availability

The data presented in this study are available on request from the corresponding author. The data are not publicly available due to institutional research policies.
